# NASA’s Cold Atom Lab (CAL): system development and ground test status

**DOI:** 10.1038/s41526-018-0049-9

**Published:** 2018-08-21

**Authors:** Ethan R. Elliott, Markus C. Krutzik, Jason R. Williams, Robert J. Thompson, David C. Aveline

**Affiliations:** 1 Jet Propulsion Laboratory, California Institute of Technology, Pasadena, CA 91109 USA; 2 Humboldt-Universität zu Berlin, Newtonstr. 15, 12489 Berlin, Germany

## Abstract

We report the status of the Cold Atom Lab (CAL) instrument to be operated aboard the International Space Station (ISS). Utilizing a compact atom chip-based system to create ultracold mixtures and degenerate samples of ^87^Rb, ^39^K, and ^41^K, CAL is a multi-user facility developed by NASA’s Jet Propulsion Laboratory to provide the first persistent quantum gas platform in the microgravity conditions of space. Within this unique environment, atom traps can be decompressed to arbitrarily weak confining potentials, producing a new regime of picokelvin temperatures and ultra-low densities. Further, the complete removal of these confining potential allows the free fall evolution of ultracold clouds to be observed on unprecedented timescales compared to earthbound instruments. This unique facility will enable novel ultracold atom research to be remotely performed by an international group of principle investigators with broad applications in fundamental physics and inertial sensing. Here, we describe the development and validation of critical CAL technologies, including demonstration of the first on-chip Bose–Einstein condensation (BEC) of ^87^Rb with microwave-based evaporation and the generation of ultracold dual-species quantum gas mixtures of ^39^K/^87^Rb and ^41^K/^87^Rb in an atom chip trap via sympathetic cooling.

## Ultracold atomic physics: space station bound

US scientists are developing and testing an instrument for trapping and cooling ultracold atoms in preparation for the launch of the device to the International Space Station (ISS). Quantum mechanical effects are enhanced at temperatures near absolute zero, and the microgravity conditions of the ISS will allow atom traps to decompress to a new regime of picokelvin temperatures and ultra-low densities. David Aveline and colleagues from the Jet Propulsion Laboratory at the California Institute of Technology present a status of the Cold Atom Lab (CAL) instrument’s ground development and test progress. The team demonstrates the system capabilities by creating Bose-Einstein condensates of rubidium atoms with microwave-based evaporative cooling and quantum gas mixtures of rubidium and potassium in a magnetic trap formed by current carrying wires on a compact chip.

## Introduction

The thermal, random motion of atomic gases fundamentally limits their free space measurement time and restricts their manipulation. Forty years of breakthroughs in the creation of ultracold quantum gases have resulted in standardized cooling techniques and technologies that are mature to the point of supporting large scale dedicated facilities,^1–3^ portable field-ready devices,^4–6^ and commercially offered components.^7–9^ In recent decades, national consortia and experimental groups seeking still colder temperatures and extended measurement times have successfully pushed ultracold atoms and related technologies into the free fall conditions of drop towers,^10–12^ zero-g airplanes,^13,14^ and sub-orbital launch vehicles.^15–18^ Here, two substantial benefits are realized. The first is access to a new parameter regime of quantum gases at picokelvin temperatures and ultra-low densities. Broadly, this is achieved by removing the asymmetric tilt that gravity introduces along one direction of the confining potential used to trap and cool atoms. It is then possible, for example, to decompress these traps far beyond what is achievable on the ground. Secondly, the wave nature of ultracold atoms released from these traps is the basis for inertial sensing atom interferometers, the precision of which scales as the square of their free space evolution time. Recent milestones of these ruggedized, autonomous devices operating in reduced gravity include the generation of Bose–Einstein condensates (BEC) and the demonstration of matter-wave interferometry.^19^ These pathfinders underline both the technical feasibility and the future potential for high-precision fundamental physics applications including searches for dark energy,^20^ quantum tests of Einstein’s equivalence principle,^21,22^ and long-baseline gravity wave detectors.^23,24^

The persistent free fall condition of low Earth orbit is recognized as the next natural destination for cold atom work to take advantage of microgravity. Proposals include both satellite-based missions^21,25–28^ and experiments to operate aboard the International Space Station (ISS).^29–34^ The ISS becomes particularly attractive when compared to a satellite mission both in terms of cost and the constant human presence, which enables instrument modifications and upgrades. Leveraging these advantages, the Cold Atom Lab (CAL) is designed to provide the first ultracold quantum gas experiment aboard the ISS by utilizing an apparatus developed, assembled, and qualified by NASA’s Jet Propulsion Laboratory (JPL).^35^ CAL is intended to operate as multi-user facility for principal investigators to remotely study ultracold and quantum degenerate samples of ^87^Rb, ^39^K, and ^41^K, including dual-species mixtures of Rb and K. In this article, we report the system development and test status of the CAL instrument, highlighting the successful production of dual species ultracold gases in CAL’s ground testbed facility.

Conceptually, the CAL system consists of three primary subsystems: the science module, electronics, and the laser and optical distribution system. In order to support reliability, repair, integration, and replacement of components, all three subsystems leverage commercially offered components in an architecture intended to be compact, simple, and modular. This modular design has not only allowed parallel development of different subsystems but also various versions of the same subsystem. In particular, CAL’s ground testbed currently supports multiple physics packages, which are based on a commercially available dual-cell vacuum chamber featuring an atom chip.^36^ CAL’s ground testbed has also developed two distinct “science modules,” the project designation for physics packages prepared specifically in the flight configuration of robustly integrated optics, magnetic coils, two CMOS cameras, microwave and radio frequency (RF) emitters, a water-based cooling loop, and magnetic shields. Details of the science module and physics package can be found in the “Materials and Methods” section of this paper. This modular architecture also allows more readily available prototype/ground support systems of optics and electronics to test the functionality of the science modules during development of the flight models. Specifically, all degenerate and ultracold atom results reported in this article use the flight science module, flight equivalent lasers/optical fibers, and ground support electronics as the flight electronics were finalized. The electronics described in Materials and Methods reflect these ground versions.

## Results

We have successfully produced degenerate samples of ^87^Rb and ^41^K in addition to nearly degenerate ^39^K in the atom chip magnetic trap within CAL’s flight science module. Beginning with the loading of the atom chip trap from dual species ^87^Rb/^39^K or ^87^Rb/^41^K in 3D and 2D magneto-optical traps (MOTs), the production of a ^87^Rb BEC follows using either RF or microwave-induced evaporation though the selective rejection of the most energetic ^87^Rb atoms via transfer from the low magnetic field-seeking |*F* = 2, *m*_*F*_ = 2〉 state to a high-field-seeking, anti-trapped state. As described in the section “Materials and Methods,” our procedure loads a limited number of K atoms into the chip trap and cannot afford the atom losses inherent to removing the hottest potassium atoms through a direct evaporative process. Instead, we evaporate only ^87^Rb, which in turn sympathetically cools trapped potassium. This cooling is accomplished specifically with a microwave evaporation scheme, transferring the most energetic |22〉 ^87^Rb to the anti-trapped |11〉 state, a state transition that is separated by more than 6.8 GHz.^37–45^ Because the spacing of the similar hyperfine states in ^41^K and ^39^K are 254 MHz and 462 MHz, respectively, microwave signals at 6.8 GHz address only ^87^Rb. This is in contrast to a more typical RF evaporation scheme, which instead operates on states with frequency spacings that are almost exactly the same in ^87^Rb and either species of bosonic potassium, specifically transitioning trapped |22〉 atoms to the more weakly trapped |21〉 state before arriving at the non-magnetic |20〉 state or anti-trapped |2−1〉 state. However, it follows that microwave evaporation yields the comparative disadvantage of not immediately removing any |21〉 ^87^Rb atoms, should they also exist in the trap. The source of the |21〉 impurity during microwave evaporation of |22〉 ^87^Rb has been suggested to result from either inelastic spin-changing collisions^37,39^ or an ejected |11〉 atom moving to a point of higher magnetic field where the microwave knife becomes resonant with the |11〉 to |21〉 transition.^38,40,44^ Consistent with either of these possible cases is that the |21〉 impurity is observed to emerge during the evaporation process, and not simply as the result of inefficient optical pumping. If not addressed, these |21〉 atoms can act as an additional heat load to be sympathetically cooled or potentially remove potassium atoms via energy released from interspecies collisions that do not conserve electronic spin states. Our microwave evaporation protocol incorporates five linear frequency ramps that at low temperatures are interspersed with brief clearing stages at a second fixed frequency (6.846 GHz) resonant with the ^87^Rb |21〉 to |11〉 transition at the 8.4 G trap bottom to recurrently remove the unwanted |21〉 atoms. Beginning 60 MHz above the bare hyperfine transition at 6.834 GHz, these five ramps together last a total of 1.5 s, with up to one hundred equally spaced jumps to 6.846 GHz.

**Fig. 1:**
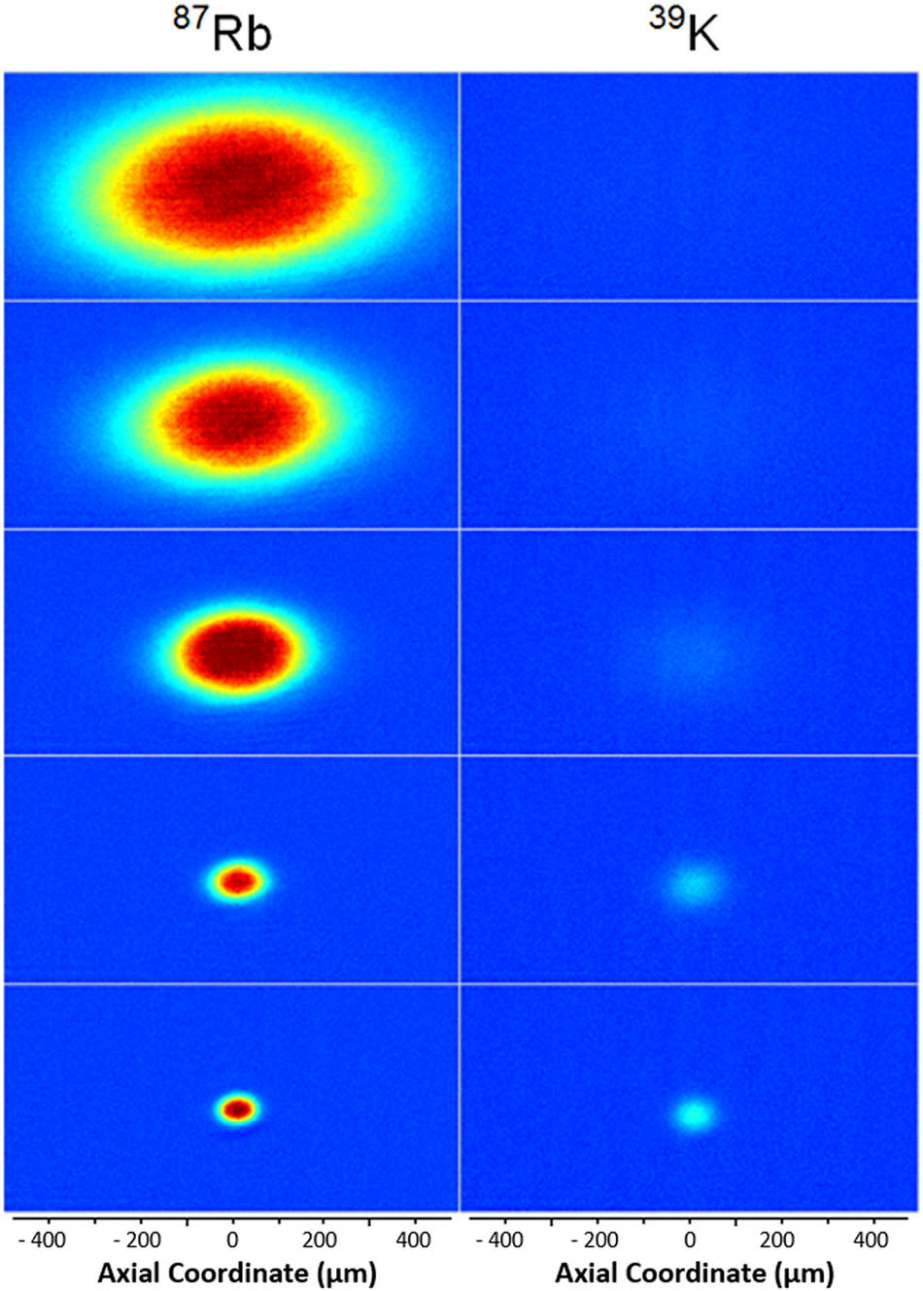
False color absorption images of ^87^Rb and ^39^K after 3 ms of expansion from an atom chip trap at five different stages of microwave-induced evaporation of rubidium and the corresponding sympathetic cooling of potassium. Higher temperature/atom number data are shown for illustrative purposes. The ^39^K atoms loaded onto the chip are initially so diffuse that our imaging fails to detect them. The last stage shows 70,000 ^39^K atoms at a temperature of 1.6 μK and phase space density (PSD) of 0.05. ^87^Rb numbers in the final stage are 155,000 atoms at a temperature of 1.6 μK and a PSD of 0.025

Following evaporation, we decompress the trap via a 70% reduction of the the bias fields in 100 ms before release and imaging. Initial results of sympathetically cooling ^39^K with ^87^Rb yield ^39^K temperatures of 170 nK with 1.1 × 10^4^ atoms and a phase space density (PSD) of 0.6. For illustrative purposes, Fig. 1 shows a higher temperature/atom number example through five stages of evaporation. Additionally, our system has cooled ^41^K to degeneracy, with current results yielding an 11% condensate fraction out of 4200 total atoms at 76 nK and a PSD of 3.5. The larger collisional cross section of ^41^K/^87^Rb compared to ^39^K/^87^Rb increases not only the comparative evaporation efficiency, but also yields more effective sympathetic cooling during the initial loading of the magnetic quadrupole trap. Repeating the above procedure without loading potassium produces ^87^Rb BECs with 3 × 10^4^ total atoms and a condensate fraction of 10%, comparable to the performance of our ^87^Rb BECs formed using standard RF evaporation (5 × 10^4^ atoms with 10% BEC fraction). To the best of our knowledge, these results mark the first use of an atom chip to evaporate ^87^Rb to condensation using microwaves, sympathetically cool either bosonic potassium isotope, and produce degenerate ^41^K.

**Fig. 2:**
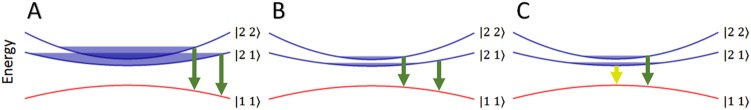
Necessity of two frequencies to remove the |21〉 impurity during microwave evaporation of |22〉 ^87^Rb to BEC in a harmonic magnetic trap with trap bottom *B*_0_. If a |21〉 population exists in thermal equilibrium with |22〉 atoms, it will form a cloud with a larger mean-square cloud size than the |22〉 cloud due to weaker coupling to the magnetic field. (**a**) Maximum total energy of a trapped atom in either state exceeds *μ*_B_*B*_0_, where *μ*_B_ is the Bohr magneton, and a single frequency (green arrow) that evaporates the most energetic |22〉 atoms to the anti-trapped |11〉 state will also remove the hottest |21〉 atoms. (**b**) The maximum total energy is less than *μ*_B_*B*_0_, and the single |22〉 evaporation frequency is too high to affect the |21〉 atoms. (**c**) A second frequency (yellow arrow) resonant with the |21〉 to |11〉 transition at *B*_0_ removes all trapped 21 atoms

While the first group to demonstrate interspecies sympathetic cooling to a BEC did not include techniques to directly remove |21〉 ^87^Rb atoms,^43^ their subsequent implementation of a countermeasure against the |21〉 state increased evaporation efficiency to allow the first mixture of dual species BECs.^45,46^ All later implementations of ^87^Rb microwave evaporation have incorporated an additional frequency targeting the |21〉 atoms throughout the entire evaporation ramp, either continuously operating^38–45^ or repeatedly pulsed.^37^ As noted, CAL employs a variation of the latter technique. However, at higher temperatures, we find it is possible to evaporate sufficiently with only a single frequency. We note that this is due to a combination of two effects that exist for |21〉 and |22〉 atoms simultaneously confined in a harmonic magnetic trap with trap bottom *B*_0_. First, a |21〉 population is more weakly coupled to the magnetic field, experiences a looser confinement, and when thermal equilibrium is assumed, forms a larger cloud compared to a |22〉 population. Secondly, a |21〉 atom at the same magnetic field as a |22〉 atom requires a lower frequency microwave field to evaporate to the |11〉 state. Quantitatively, it is easily shown for this case that if a |22〉 atom with total energy *E* relative to its trap bottom can climb the trap wall to a magnetic field of *B*, related by *E* = *m*_*F*_ *g*_L_ *μ*_B_(*B* − *B*_0_) where *μ*_B_ is the Bohr magneton and *g*_L_ is the Landé g-factor, then a |21〉 atom with the same total energy *E* can reach a higher magnetic field given by 2*B* − *B*_0_. If the most energetic |22〉 atoms reach *B*, a microwave field with frequency *ω*_*hf*_ + 3*Bμ*_B_/(2ℏ), where *ω*_*hf*_/2*π* is the zero field ^87^Rb hyperfine splitting, is required to transition them to the |11〉 state. Comparison to the frequency that evaporates the most energetic |21〉 atoms, given by *ω*_*hf*_ + (2*B* − *B*_0_)*μ*_*B*_/ℏ, yields a critical microwave frequency, *ω*_*c*_ = *ω*_*hf*_ + 3*B*_0_*μ*_*B*_/ℏ, above which a single frequency is sufficient to evaporate |22〉 atoms while still decreasing the |21〉 population, but below which a second frequency becomes necessary to remove the remaining |21〉 impurity (Fig. 2). Similarly, there is also a critical total energy of a trapped atom in either state relative to its respective trap bottom given by *μ*_B_*B*_0_. Following this simplified model, a harmonic magnetic trap with a higher trap bottom would therefore have a relatively larger region where two frequencies are necessary for efficient microwave evaporation.

## Discussion

Following the work presented here, one science module will remain at JPL facilities for PI-specific experiment development and further optimization of dual species quantum gas production, while the primary science module is integrated into the fight instrument for acceptance testing and final ground verification as an autonomous instrument before delivery to the launch site. Following CAL’s arrival at the ISS, there will be an initial installation phase by the onboard crew, including its first power up and basic communication tests between the instrument and the Ground Data System (GDS). In the subsequent commissioning phase, the flight system performance will be tested and optimized for microgravity with the option of data downlinked to the GDS in real time. In the nominal operation phase, the CAL instrument will be run with a duty cycle of 1 min for up to 8 h per day during crew sleep, to minimize vibrations. These operations will be conducted through a collaboration of the CAL PIs and the CAL team based at JPL, with each PI allocated schedule for their experiments to be run. The proposed research will investigate applications of atom interferometry for future precision measurements in the areas of Earth observation and fundamental physics,^47,48^ Feshbach resonances to control differential center-of-mass distributions of dual-species quantum gas mixtures,^49,50^ bubble-shell geometries for Bose–Einstein condensates,^51^ as well as few-body systems in new temperature and density regimes that are prerequisites for the next generation of Efimov experiments.^52^ After the conclusion of these experiments, the instrument will either enter a decommissioning phase or, through the use of orbital replacement units, receive novel, upgraded capabilities for extended mission operation and new microgravity experiments of advancing scope and complexity.

## Materials and methods

### Science module

**Fig. 3:**
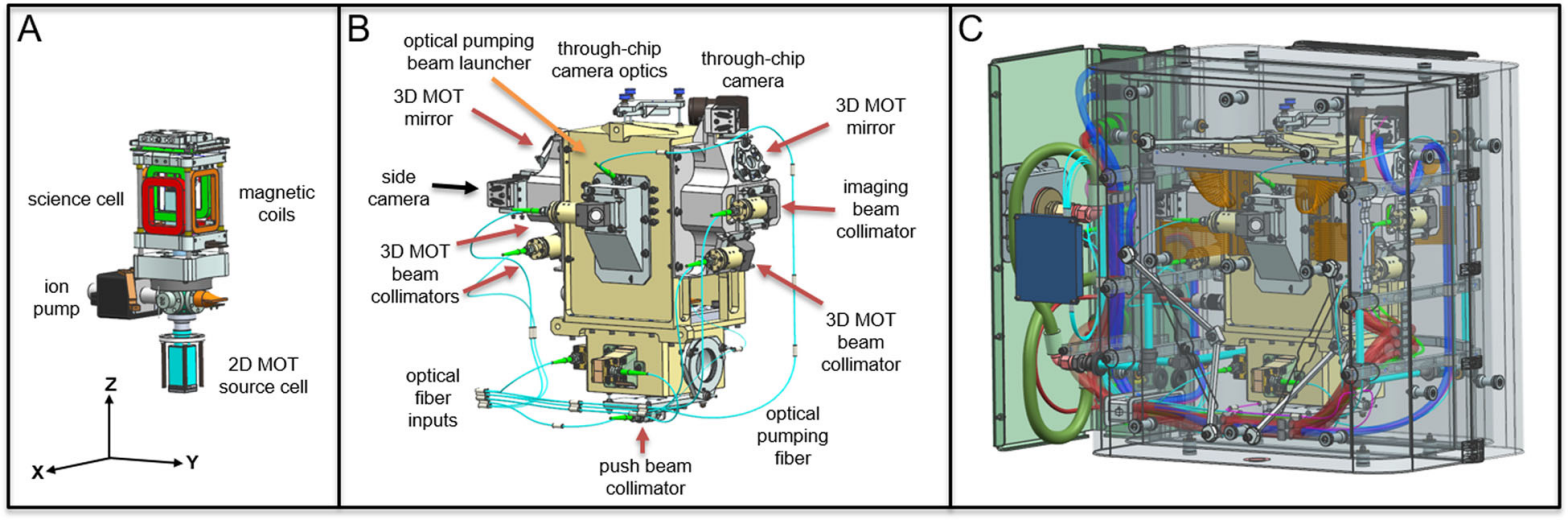
Layered overview of the CAL science module. (**a**) The physics package and magnetic coil assembly. MOT coils are shown in red, with the central axis of coils oriented along the x-axis. Transfer coils (in green) are vertically offset from the MOT coils. (**b**) Aluminum enclosure for the physics package securing the cameras and optical fiber collimators. Collimators and mirrors are emphasized in order to illustrate beam geometry, with red/orange arrows indicating effective directions of beam propagation. Two sets of 3D-MOT beams propagate diagonally in the *y*–*z* plane, while the third propagates along the *x*-axis through the MOT coils. Input for the optical pumping beam path (orange arrow) is positioned above the *x*-axis MOT beam collimator. The collimators opposite the two cameras provide light for absorption imaging, while the collimator below the through-chip camera also provides the push beam. The remaining two collimators surrounding the source cell send light to the 2D-MOT. Opposite the 2D- and 3D-MOT beam collimators are retro-reflecting mirrors (the retro-reflecting mirror for the *x*-axis MOT beam is not visible from this orientation). (**c**) The outside of this aluminum structure is bracketed to the water cooling loop and further enclosed by the magnetic shields with feed through connections from the other subsystems of the CAL instrument. Fully assembled, the dimensions are 46 cm × 30.5 cm × 58.5 cm, at a mass of 45 kg

The physics package resides inside CAL’s science module, which also houses all the hardware that maintains the appropriate local environment of magnetic fields, electromagnetic fields (optical, RF and microwave), as well as thermal and mechanical stability (Fig. 3). Forming the outer boundary of the science module is a dual layer magnetic shield, which mitigates the significant and regularly modulated external magnetic fields experienced on the ISS. A bulkhead on the side of shield holds all necessary optical, electrical and thermal feedthroughs for the physics package, including the water cooling loop, seven optical fibers (780 nm cooling, 780 nm repump, 767 nm cooling and repump, imaging, optical pumping, 2D-MOT push, and Bragg beam), power for the magnetic coils, atom chip currents, alkali metal dispensers, ion pump, two cameras (plus datalinks), and RF/microwave signals.

CAL’s physics package (Fig. 3a) is based on ColdQuanta’s commercially offered RuBECi vacuum chamber,^7,36^ modified for flight and CAL-specific science objectives. These modifications include a second atomic source for potassium, specialized anti-reflective coatings, a unique configuration of atom chip conductive paths, as well as features for improved electrical, thermal, and mechanical integrity. The physics package consists of a dual glass-cell vacuum chamber, with one cell (source cell) housing two 12 mm alkali metal dispensers (AMD, SAES) and a non-evaporable getter (NEG, SAES ST175), while the second (science cell) holds an ultra-high vacuum with an atom chip forming its ceiling. In order to maintain pressures below 10^−10^ Torr in the science cell, a miniaturized 2 L/s ion pump and a second NEG are implemented in a six-flange stainless steel vacuum intersection. The source cell is surrounded by four rods lined with permanent magnets for the production of overlapped dual species 2D-MOTs. A smooth silicon chip with a 0.75 mm diameter pinhole provides differential pumping with the science cell. This pinhole also allows the propagation of cold atoms from the 2D-MOT in the source cell to the 3D-MOT in the science cell. A push beam is directed along the axis of this pinhole from below the source cell, while partial reflection of this beam’s edges from the silicon surface additionally boosts the loading rate of the 3D-MOT.^36,53^

The CAL science cell is surrounded by ten rectangular-shaped magnetic coils, housed in an anodized aluminum structure that couples to the water cooling loop via eight thermal straps. These enclosed coils produce the fields necessary for the MOT, magnetic transport, transfer into a chip trap, and tuning near Feshbach resonances. The coils are capable of generating magnetic bias fields along each Cartesian axis. Along the *x*-axis, independent coil control allows anti-Helmholtz configurations for magnetic trapping gradients. Two separate pairs of vertically offset x-coils translate the zero-field position from the MOT location to the atom chip. Incorporated into these upper x-coils (the transfer coils) are two turns of wires designated as the fast Feshbach coils (FFC). In conjunction with the transfer coils in Helmholtz configuration (producing up to 325 G), the FFC ramps the x-bias field by 3G within 50 μs. The upper side of the coil housing attaches to a breakout board for electrical connections to the atom chip, a microwave antenna, and a RF antenna. This breakout board also concentrically secures these loop-style antennas a few mm above the atom chip (Fig. 5).

**Fig. 4:**
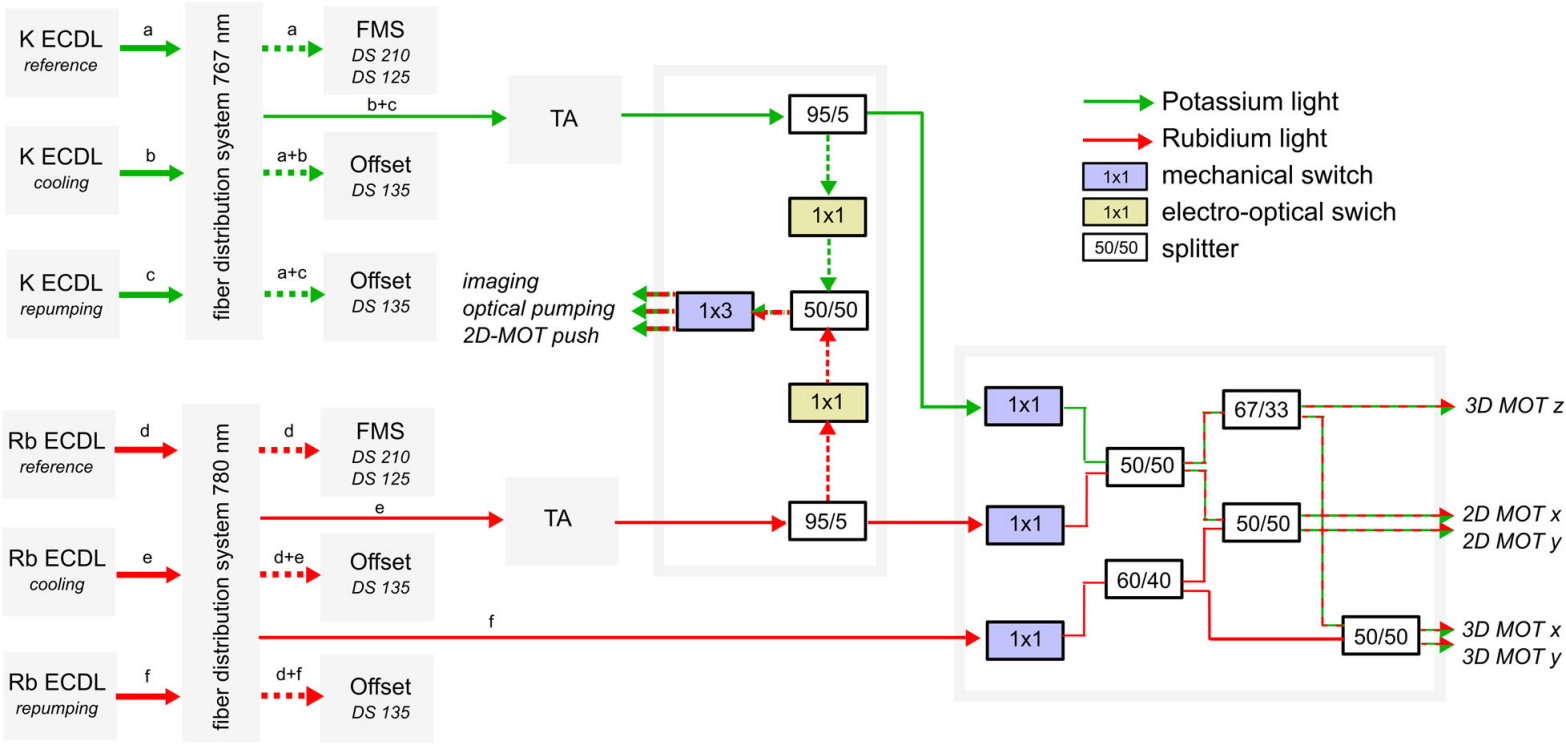
Schematic of the CAL laser system for dual-species MOT operation, state preparation, and absorption imaging. Laser light is sourced from external cavity diode lasers (ECDL), with a reference laser for each species, labeled (**a**) for K and (**d**) for Rb, locked to an atomic line via frequency modulated spectroscopy (FMS). The potassium cooling (**b**) and repump (**c**) light is amplified in the same tapered amplifier (TA), where we have observed no intensity fluctuations from potential mode competition. For rubidium, only the cooling light (**e**) seeds the 780 nm TA, with the Rb repump laser output (**f**) propagating without amplification. The cooling and repump light for Rb and K is recombined and directed to both the 2D- and 3D-MOTs, while a switching assembly directs additional light to either the 2D-MOT push beam, the optical pumping path, or the imaging path. This fiber path selection is determined by high-extinction fiber coupled LEONI mechanical switches on the sub-ms timescale, while short pulse generation is accomplished with Agiltron Nanospeed electro-optical switches. All switches are fiber coupled. The n × m notation is [number of switch inputs] × [number of switch outputs]. The majority of our light losses occur through the switches, which are about 70% efficient. Per species, the 3D-MOT and 2D-MOT path provides up to 20 mW and 30 mW, respectively, at collimated outputs within the science module. The switch that distributes light to the imaging, optical pumping, and push beam, produces up to 2 mW of light from its output per species. This full power is used for optical pumping, and attenuated to 0.2–0.9 mW at the output paths for imaging and push beams. The flight system includes a seventh ECDL at 785 nm (not pictured) to be combined with a future, on-orbit upgrade to the physics package, providing the additional capability of Bragg interferometry

**Fig. 5:**
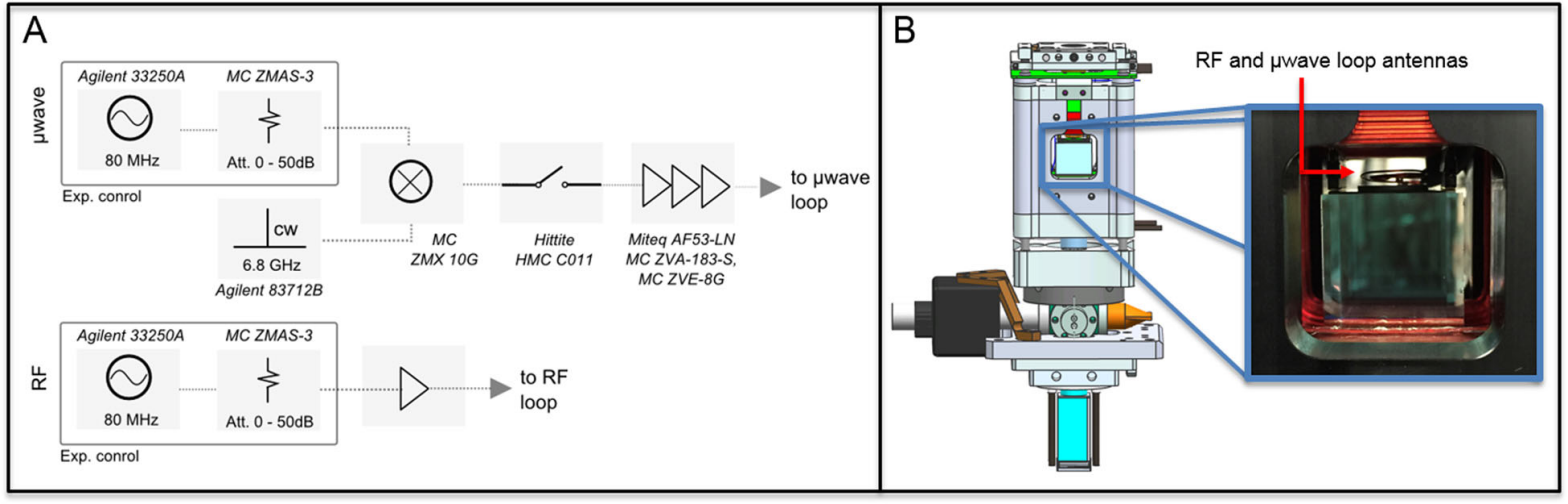
(**a**) Layout of the ground test equipment comprising the microwave and radio frequency (RF) chain for forced evaporation of ^87^Rb. Tunable RF signals are generated by a 80 MHz arbitrary waveform generator (AWG) and sent through a voltage-controlled attenuator and an amplifier. A tunable microwave source results from mixing a separate AWG and voltage-controlled attenuator with the output of a synthesized CW generator. We implement a fast and high-extinction ratio microwave switch followed by a chain of amplifiers. (**b**) Physics package science cell with loop antennas positioned millimeters above the atom chip assembly. The RF emitter is a 10 mm-diameter double loop of wire radiating 1–50 MHz, and the microwave antenna is a 12.5 mm-diameter single loop of wire operating within 100 MHz of 6.834 GHz. The capabilities of this ground test equipment allow us to set a fixed carrier wave at 6.846 GHz, the frequency resonant with the ^87^Rb |21〉 to |11〉 transition at a 8.4 G trap bottom to constantly remove unwanted |21〉 atoms while evaporating uninterrupted with the upper side band. The CAL flight electronics, however, produce one pure tone. We therefore adopted our procedure to follow the capabilities of the flight instrument

Within the science cell, we have obtained a 10 s lifetime in the tight trap used for forced evaporation, where (*ω*_*x*_, *ω*_*y*_, *ω*_*z*_) = 2*π* × (300, 1450, 1490) and a variable hold time is introduced following three evaporation stages. A variable hold time in the same trap following BEC production yields a condensate lifetime of 2 s. Limitations to the trap lifetime result from noise on the current drivers sourcing the currents for the magnetic trap (both the chip traces and coils) and vacuum quality. The flight electronics exceed the performance of ground-use commercial chip current drivers, as the ground chip drivers achieve a lifetime of 8 s in the same configuration. We find that the lifetime is strongly influenced by the operation of the dispensers within the source cell, although any related change in vacuum quality is below the range of the ion pump gauge. Continuous operation of both dispensers will gradually degrade the lifetime over a period of 8 hours, with nominal performance routinely restored after 16 hours of off time.

External to the physics package and coil housing is an aluminum structure that provides mechanical stability for collimators, mirrors, and two cameras relative to the physics package. Each camera is a compact CMOS unit (Basler ACE acA2040-180 kmNIR) with 2048 × 2048 pixels, 5.5 μm pixel size, a quantum efficiency of 35%, and a dynamic range of 10 Bits. Both cameras are thermally strapped to the water cooling loop. We label the camera positioned to the side of the science cell that views the *y*–*z* plane perpendicular to chip surface as the “side camera,” and the camera secured with imaging optics above a 3 mm circular window integrated into the chip surface as the “through-chip camera.” For a typical trapped cloud located a few hundred microns below the atom chip surface, the diameter of this window provides a potential numerical aperture up to 0.9.^54^ The MOT beam collimators are provided by Schäfter + Kirchhoff and feature a titanium housing in addition to integrated clean up polarizers and quarter wave plates. Four of these collimators produce 12 mm-diameter beams for the 3D-MOT and side camera imaging, and two more collimators at the level of the source cell produce 2D-MOT beams with an elliptical cross section of 12 mm by 24 mm. Opposite the 2D- and 3D-MOT beam collimators are retro-reflecting mirrors, doubling the respective powers as seen by the atoms. As shown in Fig. 4, the optical distribution system provides the same cooling and repump frequencies for each species to both the 2D- and 3D-MOTs. A 4 mm diameter collimator at the bottom of the source cell directs a vertical beam through the center of the physics package to serve as either the push beam or the absorption imaging beam for the through-chip camera. In order to provide efficient optical pumping, an additional dedicated output coupler and quarter wave plate on the side of the science cell produces a slightly diverging beam that is roughly 1 cm in diameter at the location of the atoms.

### Laser and optical distribution system

The optical distribution system of CAL, on the ground and in flight, is based on commercially available lasers and components to create an all optical fiber-based distribution system (Fig. 4). We operate one laser system for both bosonic potassium isotopes at 766.701 nm, and an additional system for ^87^Rb at 780.24 nm. Laser cooling light is sourced by external cavity diode lasers (New Focus Vortex Plus) with a ruggedized design for flight. Each laser features an internal 30 dB optical isolator and pigtailed optical fiber output coupler. The output light is guided through single-mode polarization maintaining fibers (Corning PM 850) and fiber splitter arrays from Evanescent Optics.

For each atomic element, we use three separate lasers and a tapered amplifier (TA, New Focus TA-7600). One reference laser is stabilized to a temperature-controlled vapor cell module (Vescent D2-210) via saturated absorption spectroscopy. The other two lasers are stabilized via frequency offset locks to the *D*_2_ cooling and repump transitions. In addition to the lasers’ internal isolators, a pigtailed isolator from Thorlabs provides another 30 dB of isolation from any light back-coupled downstream of the TA input. Both TA outputs are distributed via fiber splitters and switches, providing light for 2D- and 3D- MOTs, absorption imaging, and optical pumping. All frequency adjustments are made by controlling the relative frequencies of the offset locks. While these TAs are capable of outputs up to 0.5 W, they are set to operate between 250 mW and 400 mW, for K and Rb, respectively.

### Electronics

The presently reported results utilized ground support equipment (GSE) to power and control the flight science module, in parallel with the integration of CAL’s flight electronics and software. For GSE laser operation, we use New Focus TLB-6700 tunable laser controllers, with Vescent Photonics D2-135 offset phase lock servos and a D2-125 servo providing the reference frequency lock. The TAs are each run by a New Focus TA-7600 tampered amplifier controller. The drivers for both Leoni and Agiltron fiber switches are integrated within the switch assemblies. Currents to the magnet coils are sourced from Kepco bipolar power supplies, while currents for the atom chip traces come from ColdQuanta’s two-channel atom chip driver. RF and microwave sources are shown in Fig. 5. Finally, experimental timing is accomplished with ColdQuanta's commercial computer control system. CAL’s full flight system incorporates electronics designed and tested by JPL engineers specifically for ISS compatibility.

### Dual-species ^87^Rb-^39,41^K 2D MOT atomic source, 3D MOT, and atom chip transfer procedures

Because complexity and size of the CAL instrument is reduced through the use of an all fibered distribution system, digital switches, a single tapered amplifier for each species, and overlapped MOTs, our potassium cooling strategy differs from other established techniques. We forego the sub-doppler cooling methods that require higher laser power and variable, independent intensity control of each K beam,^55,56^ as well as the direct K evaporation methods that rely on an optical trap.^42^ Instead, the primary goal of our laser cooling procedure is to maximize the amount of rubidium to sympathetically cool the available potassium.

The prioritized laser cooling of ^87^Rb closely follows the recipe described in Ref. ^36^ with K frequencies adjusted to minimize losses and heating. Once the parameters for the simultaneous loading of Rb and K into the chip trap were found, returning to the most efficient loading of Rb alone required only minor adjustments to bias fields. However, the absolute atom number and temperature is more favorable when loading Rb alone (accomplished simply by shuttering the K light sources), owing mostly to the light induced collisions in the dual species MOT. We therefore summarize our loading of the chip trap assuming the presence of both Rb and K. Generally, we also find that very similar powers and detunings from the respective cooling and repump transitions work equally well for ^41^K and ^39^K.

We begin dual species MOT loading by initially applying rubidium light only. Both dispensers in the source cell are continuously operated at approximately 2.2–2.8 A, saturating the ^87^Rb 3D-MOT within 1 s. The dual species 2D-MOT achieves an atomic flux on the order of 10^9^ atoms/s for ^87^Rb and 10^8^ for ^39^K. In order to limit the heating due to light assisted collisions, we unshutter the K light to load ^41^K or ^39^K on top of the rubidium during the last second of this loading phase. Our loading frequencies for ^87^Rb cooling and repump light are −1.5 Γ and −2.4 Γ, respectively, while the K cooling light operates at −4.8 Γ with the K repump light set at −3.4 Γ. After the dual-species MOT loading, we shutter the push beams and compress each MOT by increasing the field gradient by a factor of 2.8 from 13 G/cm and simultaneously ramping the ^87^Rb cooling frequency from −4.8 Γ to −13.2 Γ over the course of 25 ms, while the ^87^Rb repump light moves from its MOT loading value to −9.1 Γ. Here, the K cooling light also ramps slightly from −3.7 Γ to −3.2 Γ while its repumper remains fixed at −3.3 Γ. Next, we effectively zero the magnetic field for 2.3 ms of polarization gradient cooling of ^87^Rb, further detuning the ^87^Rb cooling to −28 Γ while the ^87^Rb repump light is placed at −3.3 Γ. Though we are unable to execute a parallel potassium sub-Doppler cooling protocol^55,56^ due to fixed optical intensity, we do observe a drop in the potassium temperature during this stage by sweeping its cooling light from −2.5 Γ to −1.8 Γ and fixing its repump at −2.6 Γ. Lastly, over the course of 2 ms, we optically pump Rb and K to their respective |22〉 state with 2 mW (per species) of circularly polarized light in preparation for the remaining stages of magnetic trapping. This optical pumping stage increases the number of trapped atoms by roughly a factor of 2.5. After obtaining a polarized sample, all light is shuttered while the MOT coil fields snap back on to form a magnetic quadrupole trap with a 100 G/cm field gradient at a position approximately 20 mm below the atom chip surface. The atoms are then transported to the chip by turning on the second, vertically offset pair of transfer coils to 100 G/cm while simultaneously relaxing the quadrupole trap formed by the MOT coils. The large overlap of the MOT coils and the transfer coils minimizes heating during this transport away from the MOT region. In contrast, our chip trap is non-adiabatically loaded from the quadrupole of the transfer coils using a throw and catch method. The transfer currents are chosen to match the gradient that will be formed by the final chip trap as closely as possible, before we immediately switch the transfer currents from a gradient configuration to a bias in the *x*-direction as fast as the inductance of the coils allows. This field is combined with bias fields in the *y*- and *z*-directions, and atom chip currents of 3.2 A in the main Z-trace plus 0.362 A in the dimple trace,^36^ producing a trap with trapping frequencies of (*ω*_*x*_, *ω*_*y*_, *ω*_*z*_) = 2*π* × (207, 967, 1050) Hz for rubidium, (*ω*_*x*_, *ω*_*y*_, *ω*_*z*_) = 2*π* × (310, 1450, 1570) for potassium, and a trap bottom of 8.4 G.

### Data availability

All relevant data are available from the authors.
